# Viral Load Suppression Associated With Undisclosed HIV Status Among Adolescents Aged 10 to 19 Years Living With HIV in Low- and Middle-Income Countries: Protocol for a Scoping Review

**DOI:** 10.2196/75838

**Published:** 2025-09-03

**Authors:** Mygirl Pearl Lowane, Olanrewaju Oladimeji

**Affiliations:** 1 Public Health School of Healthcare Sciences Sefako Makgatho Health Sciences University Ga-Rankuwa, Pretoria South Africa

**Keywords:** evidence synthesis, scoping review, HIV, undisclosed, viral load suppression, adolescents

## Abstract

**Background:**

HIV and AIDS remain a major health problem in sub-Saharan Africa and are particularly prevalent in children and adolescents. The prevalence of undisclosed HIV status among adolescents is also a challenge worldwide.

**Objective:**

This scoping systematic review aims to synthesize the existing literature to address viral load suppression among adolescents living with HIV who have not disclosed their HIV status in low- and middle-income countries.

**Methods:**

A comprehensive search of PubMed, MEDLINE, Google Scholar, and ScienceDirect from 2010 to 2024 will be conducted. This review will conduct evidence synthesis from the published literature on a research question regarding viral load suppression associated with undisclosed HIV status among adolescents living with HIV. The population, concept, and context framework was considered more useful in guiding this study. A random sample of 12 articles will be assigned to the 2 reviewers to pretest the data extraction tool.

**Results:**

The initial search was conducted for articles published from March 1, 2010, to December 31, 2024, and 3174 articles were returned in total. After deduplication, of the 3174 articles, 1147 (36.14%) abstracts were reviewed, and 98 (3.09%) articles have been included in full-text review. Most of the results indicated that disclosure of HIV status is positively associated with the holistic well-being of adolescents. Among adolescents, the provision of social and psychological spaces helps them vent problems, whereas behavior change interventions are essential to reduce the likelihood of unsuppressed viral load and the risk of HIV transmission among adolescents in the context of antiretroviral therapy education and counseling.

**Conclusions:**

The findings will be key in informing practices, programs, and decision-making for the integration of services for adolescents living with HIV to address the issue of HIV disclosure for the achievement of viral load suppression.

**International Registered Report Identifier (IRRID):**

DERR1-10.2196/75838

## Introduction

### Background

In recent decades, the global HIV landscape has changed, and major milestones have been achieved in both HIV treatment and prevention. Despite these significant advances, it has been reported that 28% of people living with HIV are not virologically suppressed [1]. Unsuppressed viral load (VL) is associated with an increase in the risk of AIDS progression [2]. In certain groups, the risk of nonsuppression of the HIV virus is higher [3]. HIV and AIDS remain a major health problem in sub-Saharan Africa and are particularly prevalent in children and adolescents [4].

HIV VL measurement is a key indicator in the clinical management of the HIV virus in people who are infected. Since 1996, it has been demonstrated that the VL threshold is a way of measuring the response to antiretroviral therapy (ART) and identifying patients with virological suppression or its failure [5,6]. Frequent tests are recommended as part of the management of nonsuppression and failure events, which may vary according to guidelines [7,8]. VL results are classified as undetectable (no VL detected on the test used), suppressed (≤1000 copies per mL), or unsuppressed (>1000 copies per mL) [9]. In 2023, the World Health Organization issued updated policy guidelines on the role of HIV eradication in improving health for people living with HIV and reducing HIV transmission by recommending regularly monitoring VL to detect early treatment failures. Although a threshold of >50 copies per mL was introduced, currently, the World Health Organization still considers an individual with a VL of >1000 copies per mL in 2 consecutive tests to be virally unsuppressed [7].

HIV infection in women heightens the risk of transmitting the virus to the foetus. Such vertical transmission may occur during pregnancy via the placenta, during childbirth through exposure to maternal body fluids, or after birth through breastfeeding in Walters et al [10]. The vertical transmission was mainly perinatal, whereby a mother transmitted HIV to their child during pregnancy, childbirth, or breastfeeding [11]. In 2022, approximately 1.2 million pregnant women were infected with HIV, and without treatment, between 15% and 30% of infants contracted HIV during pregnancy, labor, or birth and 5% to 15% contracted HIV during breastfeeding [12]. With an expansion of the rollout of prevention of mother-to-child transmission (MTCT) services, mothers’ VLs are likely to be suppressed through antiretroviral medication during the breastfeeding period, so the rate of MTCT of HIV could be <2% [13]. In the same year (2022), the global progress toward the elimination of MTCT was reported to be stagnated despite women with HIV receiving ART [12]. However, between 2021 and 2022, the global MTCT rates showed a slow decline after several years [14]. Several studies have highlighted that, despite efforts to reduce MTCT rapidly among HIV-exposed infants and eliminate it, the vision of a new generation without HIV has not yet been achieved [14-16].

Because of the effectiveness of ART expansions, many children living with vertically acquired HIV are now living longer to reach adolescence (10–19 years) and early adulthood [17]. By the time they reach the developmental transition period of adolescence, they have been living for a decade with a chronic disease [18,19]. Adolescents aged 10 to 19 years account for approximately 16% of the world’s population. In 2021, there were 1.7 million adolescents living with HIV, representing 5% of the population living with HIV worldwide [20]. Approximately 140,000 new HIV infections in the world were reported in 2022 among young people aged 10 to 19 years, with approximately two-thirds of all HIV infections occurring in sub-Saharan Africa, although high HIV rates were also reported in Eastern and Southern Africa [21].

The literature reveals that adolescents living with vertically acquired HIV experience poorer HIV-related outcomes, such as virologic treatment failure and poorer retention in care [22,23]. It has been highlighted that many children and adolescents living with HIV are enrolled in HIV care without knowledge of their HIV status for various reasons, such as considering that the child is still too young to understand, stigmatization, or fear of disclosing the mother’s or child’s HIV status [24,25].

HIV status disclosure is a well-known important factor determining physical and mental health outcomes for patients with HIV at any age, and it is a challenge that continues to be faced [26]. The disclosure of HIV status to children is a difficult task for parents and caregivers as it is emotional and can be linked to positive or negative health outcomes [25,26]. Care providers who delay disclosure to children identify several factors for doing so, such as concerns about negative reactions of children and inappropriate disclosure to others that may result in shame, stigmatization, and discrimination related to HIV [26,27]. Disclosure of HIV status to infected children holds several benefits, yet rates of disclosure remain low, posing challenges worldwide [28], and many infected children do not know their diagnosis even though they regularly attend clinics and receive ART [28].

HIV status disclosure is an ongoing process and can be conducted in phases. Partial disclosure to children or adolescents can take place when the child reaches the ages of 5 to 9 years, full disclosure can take place at the ages of 10 to 12 years, and psychosocial support to transition to other relevant programs can be provided at the ages of 13 to 19 years [29]. A study conducted in Uganda showed that up to 30% of HIV-infected children aged 5 to 17 years achieved full HIV status disclosure, and a similar study in Zimbabwe reported that 27% of HIV-positive children aged 9 to 15 years received full disclosure [29].

Two South African studies found that adolescents find it difficult to adhere to any ART medication regimen in the long term compared to adults. As a result, they face unsuppressed VL and virological failure [30]. With the engagement in high-risk behaviors, some adolescents who are infected with HIV fail to respond optimally to antiretroviral medications, with only 24% achieving and maintaining undetectable VLs [31]. Adolescents face distinctive challenges in taking ART because of failure to take their condition seriously, disclosure concerns, and stigma [19].

### Objectives

Although large studies on the efficacy of ART in HIV-infected adults and children have been conducted, relatively fewer data have been collected and synthesized describing how disclosure affects virological outcomes among adolescents aged 10 to 19 years on ART [31]. A scoping review is especially suitable for this topic as it enables the mapping of existing literature, identification of research gaps, and synthesis of various pieces of evidence related to the suppression of VL among adolescents with undisclosed HIV status. It might highlight gaps in knowledge about the psychological, social, behavioral, and clinical factors affecting medication adherence and VL suppression of adolescents with nondisclosed status. Given the relatively sparse focus on this subgroup between 2010 and 2024, there might be limited consolidated data and systematic reviews. Hence, this scoping review aims to systematically synthesize existing evidence on the relationship between nondisclosure and VL suppression among adolescents aged 10 to 19 years living with HIV. In addition, this review may propose personalized strategies, including initiatives to improve medication adherence and VL suppression, while respecting confidentiality and addressing stigma.

## Methods

### Overview

Scoping reviews are increasingly used as an effective and systematic method for the synthesis of knowledge [32]. It is a form of evidence synthesis that systematically determines and maps the scope of evidence available on a specific topic, field, or concept, often irrespective of the source of primary research or nonempirical evidence within or across a particular context [33]. Often, a scoping review is carried out to clarify concepts and functional definitions and inform future research [34-36]. A scoping review is particularly suitable for addressing broad research questions, to increase the understanding of the widely used approach to the search for information, but which have not yet been thoroughly examined [37,38].

This scoping review will be guided by the evidence-based methodology described by Khalil et al [39] based on the well-known framework and methodology by Arksey and O’Malley [40]. This framework comprises five phases, namely, (1) identifying the research question, (2) identifying relevant studies, (3) identifying the study selection criteria, (4) charting the data incorporating both quantitative and qualitative thematic analysis, and (5) collating, summarizing, and reporting the results.

As far as we know, no specific scoping review has been published addressing the relationship between HIV status nondisclosure and VL suppression among adolescents aged 10 to 19 years living with HIV. This proposed scoping review will address this study gap.

### Review Process

#### Phase 1: Identifying the Research Question

This scoping review will form the basis for synthesized knowledge that will present a broad overview of the evidence on this research topic. The concepts that will assist in this review will be mapped systematically and logically while searching, selecting, and synthesizing existing data [35]. The primary research question to guide this review will be the following: what is the extent and nature of evidence regarding VL suppression among adolescents aged 10 to 19 years living with HIV who have not disclosed their HIV status in low- and middle-income countries?

The secondary research questions, outlined in Table 1, will help in mapping the existing literature comprehensively while identifying gaps in knowledge about this population.

**Table 1:** Secondary research questions.

Characteristic	Research questions
Clinical and treatment outcomes	What are the prevalence rates of viral load suppression among adolescents with undisclosed status compared to those who have disclosed their status? What is the relationship between the degree of disclosure and viral load suppression outcomes?
Disclosure-related factors	What are the barriers to HIV status disclosure among adolescents and how does this impact viral load suppression? How does fear of disclosure affect treatment engagement and viral load suppression outcomes?
Psychosocial and behavioral factors	What psychosocial factors influence viral load suppression among adolescents with undisclosed status living with HIV? What role does social support play in achieving viral load suppression among adolescents with undisclosed status?
Health care system and support factors	What interventions are most effective for achieving viral load suppression among adolescents with undisclosed status? What support services are associated with improved viral load suppression in this population?

#### Phase 2: Identifying Relevant Studies for Scoping Review Eligibility

##### Overview

This scoping review will be guided by the population, concept, and context framework recommended by the Joanna Briggs Institute. The 3 elements of this scoping review eligibility framework, namely, population, concept, and context [40], will be used to guide the establishment of the inclusion and exclusion criteria. The adapted population, concept, and context framework is illustrated in Table 2.

**Table 2:** Scoping review protocol design adapted to the population, concept, and context (PCC) framework.

PCC element	Description	Operational definition
Population	Adolescents aged 10-19 y living with HIV	Any individuals aged 10-19 y who have been diagnosed with HIV and may or may not be receiving ART^a^.
Concept	Viral load suppression related to undisclosed HIV status	Viral load suppression refers to an HIV viral load of <1000 copies per mL, although thresholds may vary by national guidelines. Undisclosed HIV status involves adolescents who are unaware of their diagnosis due to nondisclosure, partial disclosure, or delayed disclosure by caregivers.
Context	LMICs^b^	The context refers to LMICs as classified by the World Bank at the time of each study’s publication, which includes health system factors, cultural norms, stigma, legal frameworks regarding consent for disclosure, and caregiver roles in adolescent HIV care.

^a^ART: antiretroviral therapy.

^b^LMIC: low- and middle-income country.

##### Synthesis of Eligibility Criteria

The inclusion and exclusion criteria to be considered for this scoping review will be based on accessible full-text published articles that are peer reviewed and in English. All quantitative, qualitative, and mixed methods studies conducted among adolescents aged 10 to 19 years living with HIV regardless of disclosure status in low- and middle-income countries will be included. The search databases will consider published data from 2010 to 2024 onward up to the time of the scoping review. The studies may be cross-sectional surveys or observational or interventional in design or include randomized controlled trials.

Any studies that do not meet the eligibility criteria, such as gray literature, systematic reviews, meta-analyses, case reports, editorials, commentaries, or opinion pieces without primary data and populations aged <10 and >19 years, will be excluded.

##### Search Strategy

To ensure a comprehensive assessment of the content, a search of PubMed, MEDLINE, Google Scholar, and ScienceDirect from 2010 to 2024 will be conducted. The authors will conduct a systematic literature search by consulting the Sefako Makgatho Health Sciences University’s online library. Keywords such as “HIV,” “disclosure,” “viral load suppression,” “adolescents living with HIV,” “adolescents on antiretroviral treatment,” and “primary caregivers” will be used for the literature search. All the identified articles meeting the inclusion criteria will be listed in the reference list. The following search strategy will be used:

[(“HIV” OR “Human Immunodeficiency Virus”) AND (“viral load suppression” OR “viral suppression”) AND (“undisclosed HIV” OR “undisclosed HIV status” OR “HIV status not disclosed”) AND (“adolescents” OR “adolescents” OR “teenagers” OR “youth”) AND (“low-middle income countries” OR “low-income countries” OR “middle-income countries”]

#### Phase 3: Study Selection

The selection process of documents will be guided by the PRISMA-ScR (Preferred Reporting Items for Systematic Reviews and Meta-Analyses extension for Scoping Reviews) model [40]. Two reviewers in this study will independently screen the titles, abstracts, and full texts of the retrieved articles to identify whether studies meet the inclusion criteria. If discrepancies arise and they fail to reach consensus through discussions, an independent reviewer to mediate the situation will be considered. The results of the search will be presented in a PRISMA-ScR flow diagram.

#### Phase 4: Charting the Data

Data from eligible studies will be charted independently by 2 researchers using a standardized data extraction tool. The chart will capture the relevant information using key study terminology and characteristics, such as the participant demographics, study objectives, study setting, population, and key results of the studies reviewed. This chart will be used to capture such information for each article [41]. The process will include reviewing the entire text to identify the variables relevant to this scoping review.

Virtual training sessions will be held for reviewers involved in the scoping review focusing on research objectives and questions, inclusion and exclusion criteria, key concept definitions, and the use of evaluation and data extraction tools. The Rayyan (Qatar Computing Research Institute) web-based software screening tool will be used to identify and remove duplicates, and the reviewers will be trained on its use.

The deduplicated results of the screening, including a unique ID, title, author, and abstract, will be evaluated in 2 phases in the Rayyan web-based software. First, the titles and abstracts of the articles retrieved from the search will be independently evaluated by 2 reviewers to determine the relevant records. Relevant records that at least one reviewer has classified as *included* or *insecure* will be moved to the second screening round. In the second phase, the complete texts of the relevant documents will be retrieved and independently checked to ensure that they meet the a priori defined study inclusion criteria.

Interrater agreement refers to the degree of coherence or agreement between ≥2 reviewers in the process of screening and data extraction. In this scoping review, a random sample covering 10% of the retrieved citations for pilot-testing will be assigned to the 2 reviewers, who will independently screen the titles and abstracts. The interrater agreement will be calculated using the Stata statistical software (version 19; StataCorp), and the Cohen κ statistical measurement will be used to assess the reliability or agreement between the 2 reviewers during the piloting phase. A κ value of ≥0.7 will be considered acceptable, indicating a substantial agreement. If the agreement is below the acceptable threshold, the criteria will be reviewed, additional training will be provided, and the pilot-testing will be repeated until a satisfactory agreement has been reached. Modification and merging will follow when an agreement is reached. Should conflict still arise, a mediator will be approached to facilitate agreement until a consensus is achieved.

#### Phase 5: Collating, Summarizing, and Reporting the Results

This scoping review will synthesize extracted data following thematic analysis for qualitative data and frequency analysis with counts and percentages for quantitative data to summarize the existing knowledge on the association between VL suppression and undisclosed HIV status among adolescents who acquired the infection through vertical transmission in low- and middle-income countries. A coding framework for qualitative studies will be developed by the 2 reviewers, whereas tables, charts, and diagrams will be used to capture data from the articles. Manuscripts will be written and submitted for peer review and for publication in a selected accredited journal. Conference posters will be designed and presented. The results will also be presented orally at relevant health care–related conferences locally and internationally. The findings will also be shared with the various stakeholders in the Department of Health in South Africa locally and nationally, including various nonprofit organizations responsible for youth-friendly services.

### Ethical Considerations

All articles that will be included in this review must provide a statement indicating that their study was subjected to ethical review and that the required ethics approval was obtained. The protocol will not be registered with PROSPERO. However, it will be published in a peer-reviewed journal.

## Results

**Figure 1:**
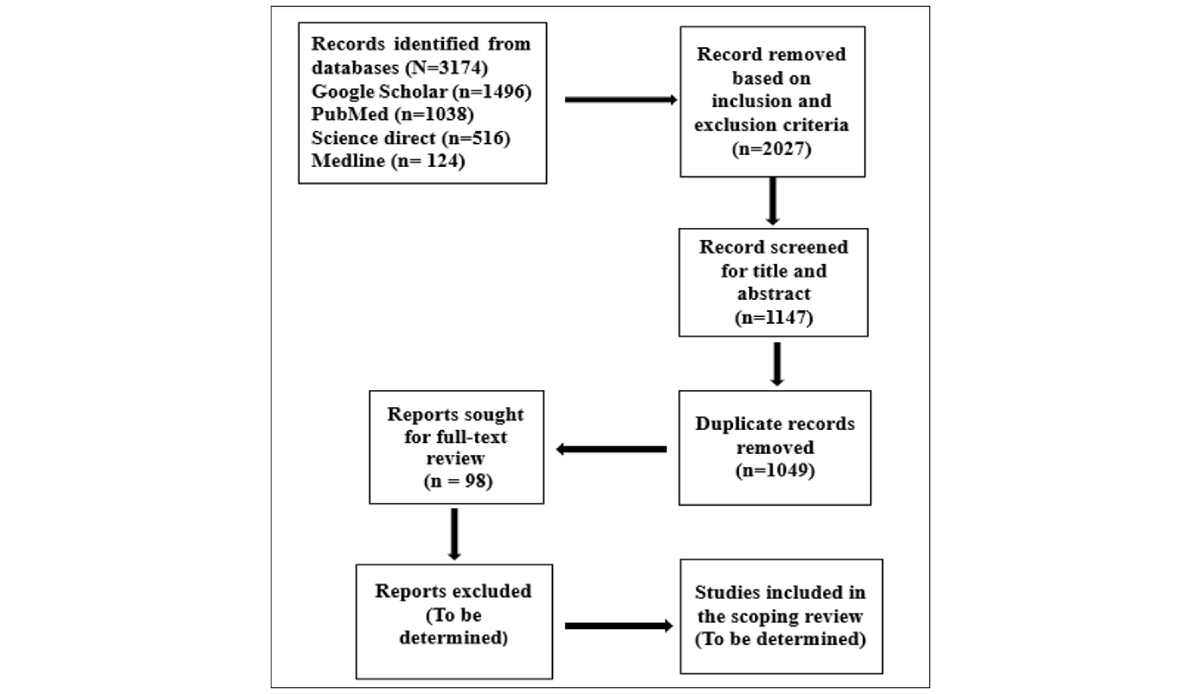
PRISMA-ScR (Preferred Reporting Items for Systematic Reviews and Meta-Analyses extension for Scoping Reviews) flow diagram example for this scoping review (self-developed).

This scoping review is currently in the study selection phase. Preliminary electronic database searches were conducted on January 3, 2025, yielding a total of 3174 records. On the basis of the inclusion and exclusion criteria, of the 3174 records, 2027 (63.86%) were excluded during this stage. After removing duplicates, 1147 unique records remained for title and abstract screening. Of the 1147 unique records, 98 (8.54%) articles have been identified for full-text review, which constitutes the next step in the selection process aimed to be completed by December 2025 (Figure 1).

Preliminary screening results show that almost 1 in 2 adolescents living with HIV were diagnosed and put to HIV care before the age of 10 years. Many adolescents living with HIV do not correctly adhere to ART because they are not informed about their HIV-positive status. The lack of disclosure causes confusion as some adolescents living with HIV think that they are correctly handling the conditions they have been falsely informed they have instead of HIV. Status disclosure has been found to positively elevate the holistic well-being of adolescents living with HIV. Moreover, disclosure of HIV status can improve quality of life, social support, immune recovery, and adherence to ART, but nondisclosure increases the risk of loss of follow-up and represents an important obstacle to achieving VL suppression. The provision of social and psychological spaces helps adolescents living with HIV vent problems, whereas HIV disclosure rules in health care facilities are key positive factors influencing the disclosure of HIV status to adolescents.

The preliminary screening results show that behavior change interventions are essential to reduce the likelihood of VL nonsuppression and the risk of HIV transmission among adolescents in the context of ART education and counseling. The results point to the importance of addressing psychosocial challenges in combination with medical treatment to facilitate adherence to ART and the achievement of VL suppression

## Discussion

### Expected Findings

This scoping systematic review aims to review and synthesize the existing literature to address VL suppression in adolescents with undisclosed HIV status; the prevalence of undisclosed HIV status among adolescents remains a challenge worldwide. Several studies indicate that poor VL suppression challenges following undisclosed HIV status vary widely among adolescents in various parts of the world [42-44].

It is imperative to review and analyze existing evidence from various parts of the world to provide a summary that will give us the full picture of the phenomenon. This scoping review will provide summary results by subgroup, such as low- versus middle-income countries, sex, and age categories, and describe factors associated with disclosure status. As adolescents aged 10 to 19 years are increasingly observed not to disclose their HIV status, which is an important factor in suppressing VL, it is necessary to deeply understand this complex problem [29,45]. Despite significant advances in HIV treatment and strategies to improve adherence, many adolescents choose not to disclose due to stigma, fear, or social pressure [46-48], which can have a negative impact on the results of VL suppression.

Most existing literature demonstrates that the probability of achieving VL suppression increases in adolescents who have an idea of their HIV status as compared to those who do not have an idea of the medication they are taking [19]. Understanding the interaction between nondisclosure and VL suppression is important to come up with appropriate interventions to meet adolescents’ social, medical, and psychological needs [3,49]. Poor VL suppression among adolescents living with HIV continues to hold back the attainment of Sustainable Development Goals and the ending of the HIV epidemic by 2030 [50-52]. Addressing these challenges will significantly improve the health status of adolescents living with HIV and contribute to the global goal of ending the AIDS epidemic.

The available evidence highlights that nondisclosure is among several factors that contribute to poor VL suppression [53]. However, the variations in factors that contribute to poor VL suppression among adolescents living with HIV are unclear in the specific context of HIV status disclosure as there is no scoping review on this topic. Full disclosure occurs when the child has reached the appropriate maturity and is prepared to fully understand HIV status [28]. In some instances, it was found that adolescent-friendly services reduce known barriers to HIV disclosure [54]. Although the evidence is not conclusive, knowing one’s HIV status and disclosure to others can improve adherence to ART. Although adolescents with HIV are faced with many situations in which they have to handle disclosure, they must learn how to cope with their own HIV status. Adolescents who better understand the issue of disclosure can disclose their status more easily than people who do not [46]. Disclosing HIV status can indirectly improve VL by enhancing compliance with treatment.

Therefore, this review will be key in providing summary findings about the prevalence of unsuppressed VL and the relationship with undisclosed HIV status among adolescent population groups. The outcomes will be key in informing health research in this field, especially research directed toward designing context-specific interventions against these challenges. It will also inform HIV and AIDS–related policies concerning VL suppression and HIV status disclosure in various settings.

### Foreseeable Limitations

This study will be limited to adolescents aged 10 to 19 years who acquired HIV vertically from their biological mothers. This scoping review will only include studies published in English. It will only cover studies published from 2010 up to the date the scoping review will be conducted. This review might be limited by the availability and quality of the data extracted from the eligible studies, which might impact the accuracy and completeness of the findings. There is a high possibility that our search strategy will omit relevant studies, and we will mitigate this risk by extending the search to multiple online databases and manually searching review articles from the institutional library.

### Conclusions

A scoping review is an emerging approach compared to systematic reviews and meta-analyses in terms of its purpose and aims. A scoping review aims to provide an insight into the available research without providing outcomes and guide clinical decision-making and interventions. A comprehensive search of PubMed, MEDLINE, Embase, Google Scholar, and ScienceDirect from 2010 to 2024 will be conducted, and the PRISMA-ScR framework will be adopted to ensure a comprehensive search of eligible articles that is rich in content relevant to the study. This scoping review will synthesize existing knowledge on HIV status disclosure and VL suppression among adolescents living with HIV regardless of whether the transmission occurred during pregnancy, during childbirth, or after birth through breastfeeding. This review will incorporate a range of study contexts to comprehensively summarize and synthesize evidence with the aim of informing practices, programs, and decision-making for the integration of services for adolescents living with unknown HIV status and facilitating the issue of HIV disclosure for undetectable VL achievement. Moreover, this scoping review might provide directions for future research priorities.

## Appendix

Multimedia Appendix 1 PRISMA-ScR checklist.

## Abbreviations

ART: antiretroviral therapy
MTCT: mother-to-child transmission
PRISMA-ScR: Preferred Reporting Items for Systematic Reviews and Meta-Analyses extension for Scoping Reviews
VL: viral load
