# Supplementary material for: Viral Load Suppression Associated With Undisclosed HIV Status Among Adolescents Aged 10 to 19 Years Living With HIV in Low- and Middle-Income Countries: Protocol for a Scoping Review
Source: JMIR Res Protoc. 2025 Sep 3;14:e75838. doi: 10.2196/75838 (PMC12444223; doi:10.2196/75838)
Supplement: Multimedia Appendix 1 [file resprot_v14i1e75838_app1.docx]

PRISMA-ScR Checklist – Multimedia Appendix

| Sections | Item | PRISMA-ScR Checklist Item | Report on Status | Page |
| --- | --- | --- | --- | --- |
| Title |  | Identify the report as a scoping review. | Title | 1 |
| Abstract |  | Provide a structured summary... | Abstract Section | 1 |
| Introduction |  | Describe the rationale... | Introduction | 2 |
|  |  | State the objectives and/or questions... | Introduction |  |
| Methods |  | Protocol and registration... | Not Registered | 6 |
|  |  | Eligibility criteria... | Methods – Eligibility Criteria | 6 |
|  |  | Search strategy... | Discussed – Search Strategy | 7 |
|  |  | Selection of sources of evidence... | Methods – Study Selection | 7 |
|  |  | Data charting process... | Methods – Data Charting | 8 |
|  |  | Data items... | Methods – Data Extraction | 9 |
|  |  | Critical appraisal of individual sources... | Not Applicable | - |
|  |  | Synthesis of results... | Methods – Data Synthesis | 9 |
| Results |  | Selection of sources... | Results – Flow Diagram | 10 |
|  |  | Characteristics of sources... | Results – Table 1 | 10 |
|  |  | Critical appraisal within sources... | Not Applicable | - |
|  |  | Results of individual sources... | Not Applicable | - |
|  |  | Synthesis of results... | Results – Main Findings | 9 |
| Discussion |  | Summary of evidence... | Discussion – Summary | 11 |
|  |  | Limitations... | Discussion – Limitations |  |
|  |  | Conclusions and implications... | Discussion – Conclusions | 12 |
| Funding |  | Describe sources of funding... | Funding - Not Applicable | - |

**
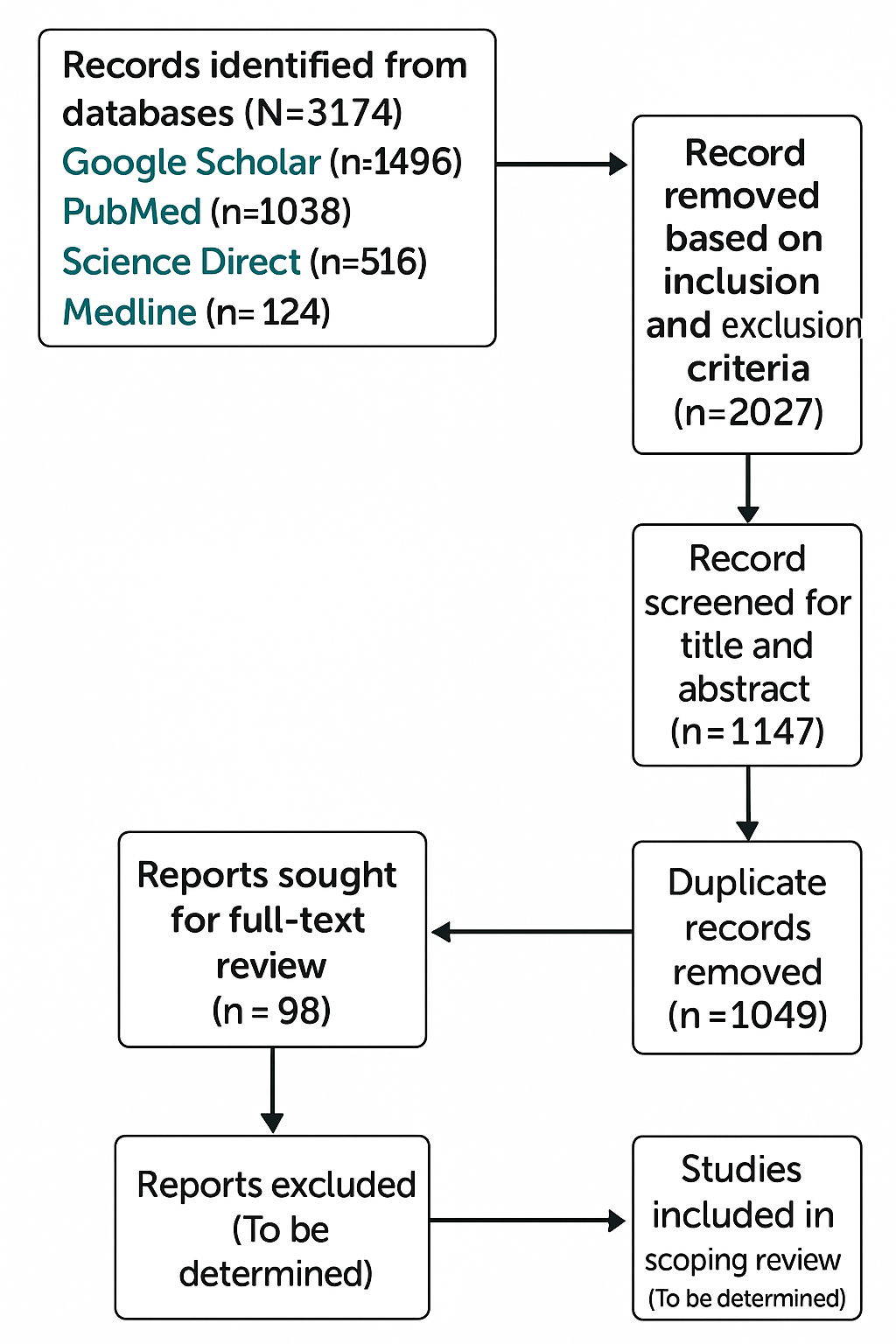
**

***Figure 1. PRISMA-ScR flow diagram example for Scoping Reviews (self-developed)***
